# Alterations of oral microbiota in patients with panic disorder

**DOI:** 10.1080/21655979.2021.1994738

**Published:** 2021-10-26

**Authors:** Zunli Xie, Weiqing Jiang, Mingzhu Deng, Wei Wang, Xian Xie, Xia Feng, Yinping Shi, Xueyan Zhang, Dong Song, Ziyu Yuan, Yonggang Wang

**Affiliations:** a Department of Neurology, The First Affiliated Hospital of Zhengzhou University, Zhengzhou, China; b Department of Neurology, Shanghai Sixth People’s Hospital Affiliated to Shanghai Jiao Tong University, Shanghai, China; c Department of Health and Medicine, Xuchang Vocational Technical College, Xuchang, China; d Department of Neurology, Beijing Tiantan Hospital, Capital Medical University, Beijing, China; e Department of Computer Science and Technology, Donghua University, Shanghai, China; f Department of Neurology, The Second Affilliated Hospital of Xinxiang Medical University, Xinxiang, China; g Headache Center, China National Clinical Research Center for Neurological Diseases, Beijing, China

**Keywords:** Anxiety disorder, panic disorder, oral microbiota, 16s rRNA sequencing, function prediction

## Abstract

The main characteristics of panic disorder (PD) include recurrent panic attacks and persistent worry, accompanied by other physical and cognitive symptoms. While recent studies have revealed that gut bacteria play an important role in anxiety and depression, little is known about the relationship between oral microbiota and PD. Therefore, the objective of this study was to explore a possible correlation between oral microbiota and PD. We conducted 16S rRNA sequencing to compare differences in the oral microbiota of patients with PD (n = 26) and healthy controls (n = 40). Patients with PD exhibited higher alpha diversity (abundance and evenness) in their oral microbiota than healthy controls, while analysis of beta diversity revealed that the two groups differed in microbial community composition. Moreover, the relative abundance of 61 genera differed between them. Overall, PD resulted in distinct oral microbial profiles that could be potential diagnostic markers and therapeutic targets.

## Introduction

Anxiety disorders are among the most common mental disorders worldwide and have a remarkable impact on the global burden of disease [1]. The 2019 Global Burden of Disease study revealed that anxiety disorders were the sixth leading cause among adolescents between the ages of 10 and 24 [2]. As one of the anxiety disorders, panic disorder (PD) has a 12-month prevalence of 2.4% and lifetime prevalence of 3.8% [3]. Moreover, patients with PD have the highest consultation rate of all anxiety disorders, incurring heavy medical costs and substantially reducing their quality of life [4–6]. PD is mainly characterized by recurrent, unpredictable panic attacks followed by persistent worry. Panic attacks as intense fear or discomfort that peaks in minutes, accompanied by other physical and cognitive symptoms (e.g., palpitations, sweating, nausea, and feelings of near-death) [7]. However, the exact pathogenesis of PD remains unclear. Possible mechanisms include genetic susceptibility, environmental factors, neurotransmitter dysregulation, as well as dysfunction of the amygdala and associated structures [8].

More and more researches are linking gut microbiota to various psychiatric and neurological disorders, including autism, anxiety, obesity, schizophrenia, Parkinson’s disease and Alzheimer’s disease [9–11]. Likewise, the mouth contains the second largest microbial community after the colon, and oral microbiota is important for maintaining human health [12]. Dysbiosis of oral microbiota can cause oral diseases such as dental caries and periodontitis [13], as well as mental diseases such as anxiety and depressive disorders [14].

In this study, we therefore hypothesized that oral microbiota should also contribute to PD. To test this hypothesis, we examined the composition of oral microbiota in PD patients via 16S rRNA sequencing. Additionally, we looked for any associations between PD and oral microbiota. Our results should provide a reference for using oral microbiota in clinical diagnosis and treatment.

## Materials and methods

### Patients

From January 2019 to January 2020, the study enrolled 26 participants with PD from neurology outpatients of Renji Hospital Affiliated to Shanghai Jiaotong University School of Medicine. All patients underwent hematology and electrocardiography examinations to rule out thyroid disease and heart disease. An experienced neurologist diagnosed them with PD according to the DSM-5 (Diagnostic and Statistical Manual of Mental Disorders, 5th edition) [15]. As a control group, 40 age- and sex-matched healthy volunteers were also enrolled. Patients were excluded if they had taken probiotics in the last month or antibiotics in the last 3 months [16,17]. A questionnaire on current medical conditions and medical history was administered to all subjects. All participants provided informed consent. This study was approved by the Renji Hospital Ethics Committee, Shanghai Jiaotong University School of Medicine (Shanghai, China).

### Sample collection

Clinical details were collected using the Patient Health Questionnaire-9 (PHQ-9) and the General Anxiety Disorder-7 (GAD-7). Patient height, weight, and age were also obtained. To collect saliva, the mouth was first washed with warm water to remove food residue. A sponge stick was then placed in the mouth for 3 min or more to fully absorb saliva without speaking or drinking during this period. The stick was then transferred to another sterile tube, immediately centrifuged, and stored at −80°C until sequencing.

### DNA extraction and sequencing

Microbial DNA was extracted from saliva using the QIAamp Fast DNA Stool Mini Kit (Qiagen, Germany), following manufacturer protocol. Total DNA integrity and concentration were determined using a Thermo NanoDrop2000 UV micro spectrophotometer and 1% agarose gel electrophoresis. Quality-checked DNA served as templates to amplify the V3-V4 region of bacterial 16S ribosomal RNA genes, employing universal primers 341 F 5′-CCTACGGGRSGCAGCAG-3′ and 806 R 5′-GGACTACVVGGGTATCTAATC-3′. Eligible DNA was paired-end sequenced with the HiSeq/MiSeq platform (Illumina, CA, USA). Long reads of the highly variable region were obtained through splicing in PANDAseq version 2.9 [18].

### Data processing

The length of 16S tags was set between 220 bp and 500 bp. Average score per read was no less than 20 (Q20), and number of N bases was no more than 3. Next, clean Reads with the same sequence were sorted according to their abundance, and Singletons were filtered out. Operational taxonomic units (OTUs) were obtained using UPARSE to cluster clean reads with 97% similarity [19]. Each OTU was assigned a representative sequence and annotated with species using the ribosomal database project (RDP) Classifier [20]. DNA sequencing and analysis were performed at the Realbio Genomics Institute (Shanghai, China).

### Statistics analysis

Clinical data were analyzed in SPSS version 25.0. Continuous variables were assessed with Student’s t-tests, while categorical variables were assessed with Pearson’s chi-square tests. Sequencing data were processed in R version 3.5.1.

Alpha and beta diversity indices reflect within-sample and between-sample diversity, respectively [21,22]. Alpha diversity indices were calculated in the quantitative insights into microbial ecology (QIIME, ver.1.9.1) [23]. For each of these indices, the rank sum test (with Wilcox. test function in R) was conducted separately to determine differences. For beta diversity, analysis of similarities (ANOSIM) was performed to test whether inter-group differences were greater than intra-group differences, thus examining whether the grouping was meaningful. Principal coordinates analysis (PCoA) was then conducted to determine the size of differences between individual samples. Subsequently, linear discriminant analysis (LDA) effect size (LEfSe) was performed to assess the effects of species abundance [24,25]. The package Phylogenetic Investigation of Communities by Reconstruction of Unobserved States (PICRUSt) was used for functional predictions of microbial communities [25]. Statistical significance was set at *p* < 0.05.

## RESULTS

We performed a cross-sectional study to compare compositional differences of oral microbiota between patients and controls via 16S rRNA gene sequencing. Oral microbial abundance and composition differed significantly between patients with PD and healthy controls. Furthermore, oral bacteria appear to influence PD via metabolic pathways.

### Characteristics of participants

The PD group did not differ significantly from healthy controls in age and sex, but their average body mass index (BMI) was higher (*p* = 0.040, Table 1). This result was consistent with previous studies, suggesting that patients with PD may have higher risks of weight gain compared with healthy controls [26,27]. Average PHQ-9 and GAD scores in the PD group were 10.0 ± 6.6 and 9.46 ± 4.95, indicating that patients might suffer from anxiety or depression.

**Table 1.** Characteristics of the PD patients and healthy controls

Characteristics	PD	HC	*p*-values
Gender			0.482
Female (%)	12(46.2%)	22(45.0%)	
Male (%)	14(53.8%)	18(55.0%)	
Age (years)^a^	41.4(12.8)	39.6(10.3)	0.572
BMI (kg/m^2^)^a^	24.1(3.0)	22.6(2. 8)	0.040
GAD-7^a^	9.5 (4.9)	-	
PHQ-9^a^	10.0(6.6)	-	

a. data are shown as mean (SD), SD: standard deviation;

PD: panic disorder; HC: healthy controls; BMI: body mass index;

GAD-7: General Anxiety Disorder-7;

PHQ-9: Patient Health Questionnaire-9.

### Microbiota diversity in patients with PD

**Figure 1.**
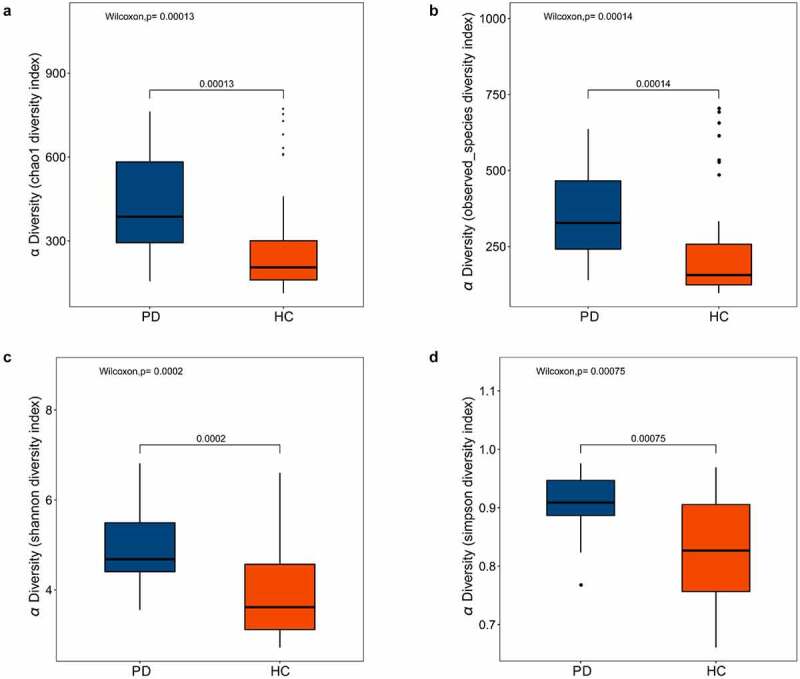
Box plots of alpha diversity indices differences between PD patients and healthy controls

**Figure 2.**
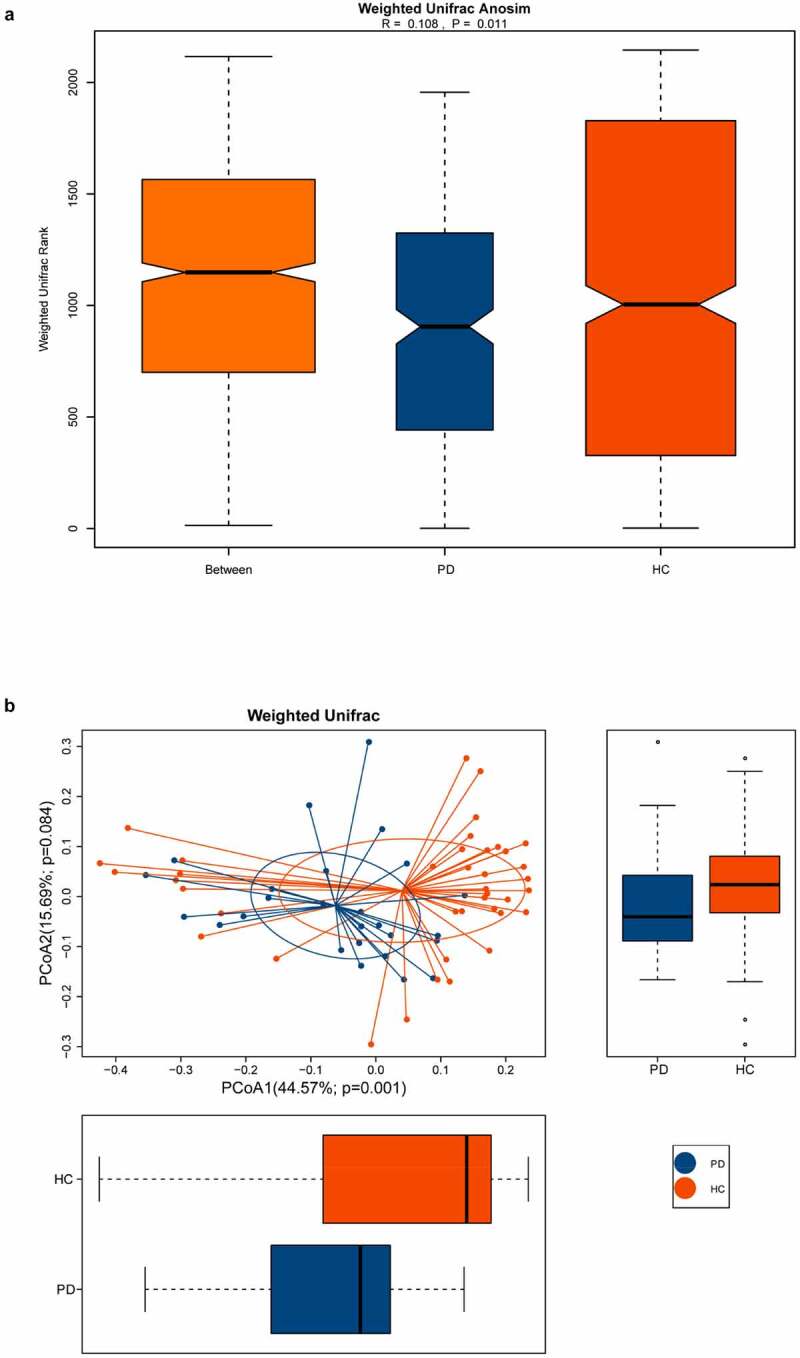
The beta diversity of the oral microbiota between PD patients and healthy controls

Various alpha diversity indices (Chao1, observed species, Shannon, and Simpson) revealed a significant difference between oral microbiota of the PD group versus healthy controls, with the former having higher abundance and evenness (Figure 1). Additionally, ANOSIM based on the weighted UniFrac algorithm (R = 0.108, *p* = 0.011), together with PCoA (*p* = 0.001), identified differences in beta diversity between samples. Thus, the oral microbiota of PD patients and healthy controls had significant differences in composition (Figure 2).

The alpha diversity indices of oral microbiota showed significant difference between the healthy controls and the PD patients. (A) Chao 1 index, *p* = 0.00013. (B) Observed species index, *p* = 0.00014. (C)Shannon diversity index, *p* = 0.0002. (D) Simpson diversity index, *p* = 0.00075. PD, panic disorder, HC, healthy controls.

The beta diversity was measured with weighted ANOSIM and weighted PCOA. (A) Based on the UniFrac algorithm, ANOSIM analysis showed that the difference among the groups was greater than the difference within the groups (R = 0.108, *p* = 0.011). (B) The horizontal and vertical axes represent the first and second principal coordinates explaining the greatest proportion of variance to the bacteria communities (showed by percentage). PD, panic disorder; HC, healthy controls; PCOA, principal coordinates analysis; ANOSIM, analysis of similarities.

### Differences in oral microbiota between PD patients and healthy controls

**Figure 3.**
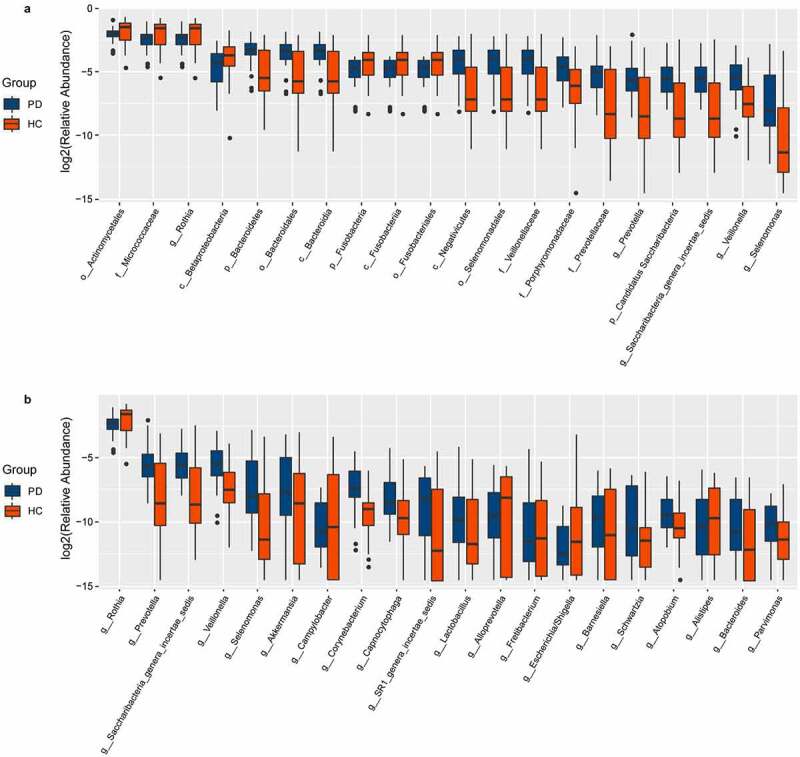
Box plots of oral microbiota differences between PD patients and healthy controls

**Figure 4.**
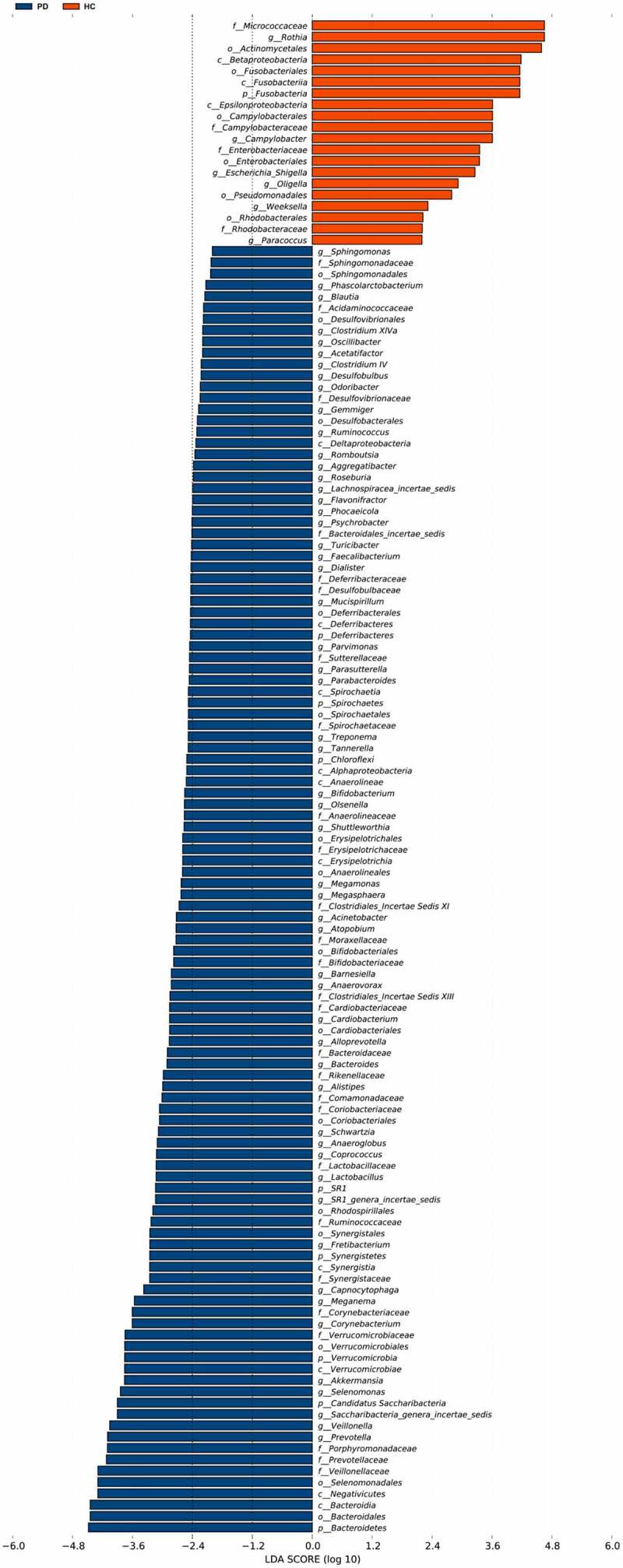
Differences of the taxa between PD patients and healthy controls

We characterized 1587 microbial taxa from 66 samples, including 1490 phyla, 1391 classes, 1353 orders, 1192 families, and 759 genera. At the genus level, we found 15 core microbes (microbiome covering 100% of the samples, Supplementary Material Table S1), with *Rothia, Saccharibacteria genera incertae sedis*, and *Veillonella* differing between patients and controls. We also identified 136 taxa with significant differences between the two groups, including 61 at the genus level (Supplementary Material Table S2, rank-sum test, *p* < 0.05). We generated bar charts for the top 20 most differentiated bacterial taxa at all levels and at the genus level, separately (Figure 3). In addition, LEfSe identified the microbiota that caused significant differences between samples (Figure 4, LAD score > 2, *p* < 0.05).

(A) Box plots of the top 20 different microbial taxa in abundance. (B) Box plots of the top 20 different microbial taxa in abundance at the general level. PD, panic disorder; HC, healthy controls; p, phylum; c, class; o, order; f. family; g, genus.

LEfSe analysis showed significant taxonomic differences in the oral microbiotabetween PD and HC groups (LDA scores (log10) > 2, *p* < 0.05). LEfSe, linear discriminant analysis effect size; LDA, linear discriminant analysis. PD, panic disorder; HC, healthy controls.

### Gene functional prediction

**Figure 5.**
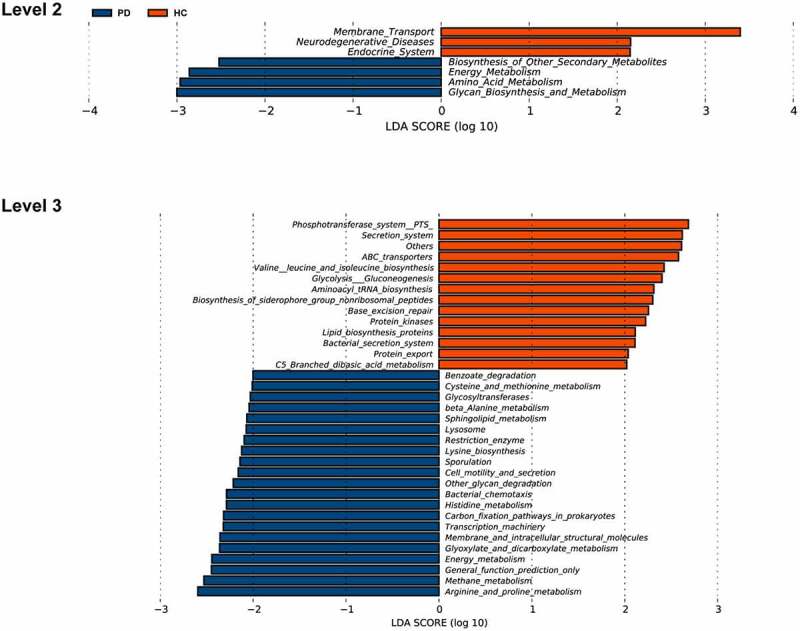
Functional predictions for the oral microbiota of PD patients and healthy controls

Based on the OTU table, we predicted oral microbial functions from the Kyoto Encyclopedia of Genes and Genomes (KEGG) using PICRUSt. The two groups differed in 29 KEGG orthologs (Supplementary Material Table S3, LDA score > 2, *p* < 0.05). Through annotations in the KEGG database, we noticed that L2-level KEGG pathways gene functions in the PD group were mainly focused on energy metabolism, secondary metabolite biosynthesis of other secondary metabolites, amino acid metabolism, glycan biosynthesis, and metabolism. In contrast, gene functions in the healthy controls related to membrane transport, neurodegenerative diseases, and endocrine system (Figure 5, LDA score > 2, *p* < 0.05).

Functional prediction of the saliva microbial genome of PD and healthy controls at the level of L2 and L3 KEGG pathways by using PICRUSt. PD, panic disorder; HC, healthy controls; KEGG, Kyoto Encyclopedia of Genes and Genomes; PICRUSt, Phylogenetic Investigation of Communities by Reconstruction of Unobserved States.

## DISCUSSION

This study is the first to identify significant differences between oral microbiota profiles of Chinese patients with PD and healthy controls.

### Oral microbiota and central nervous system (CNS) diseases

Although the effect of intestinal microbes on the brain is well-studied, we know less about how oral microbiota may influence CNS disorders. Oral bacteria can reach the brain directly and indirectly, such as via the olfactory nerve, blood, blood-brain barrier [28]. Indeed, oral bacteria such as *Porphyromonas gingivalis* and *Treponema* have been found in the brains of patients with Alzheimer’s disease [29,30]. Moreover, oral microbes can also cause inflammatory responses in the CNS. For instance, lipopolysaccharide (LPS) leakage through the blood-brain barrier in patients with autism spectrum disorder could cause CNS inflammation [28].

### Oral microbiota and anxiety

Prior research has established an association between microbes and anxiety. A mouse study, for instance, revealed that gut microbes play a role in regulating anxiety-like behaviors, and germ-free (GF) mice exhibited a decrease in such behavior [31,32]. Restraint stress experiments showed that specific pathogen-free (SPF) mice had more anxiety-like behavior than GF mice [33,34]. Furthermore, anxiety levels affected the species and abundance of the microbiota. An oral microbial and exercise study suggested that decreasing anxiety level corresponded to decreasing levels of oral microbial-causative bacteria [35]. In another study, patients with higher anxiety symptom scores also exhibited significantly more counts of oral Spirochetes and Spirochaetales than patients with lower scores [14]. Here, our findings support the hypothesis that oral microbial composition and abundance are higher in patients with PD.

### Oral microbiota and PD

We found that mean abundance of genera *Prevotella* and *Veillonella* was higher in the PD group than in the healthy controls. Relatedly, previous studies on oral microbiota and oral health found that *Prevotella* and *Veillonella* predominance in saliva may contribute to periodontal disease [13,36]. Moreover, patients with PD were three times more likely to develop periodontal disease than controls [37]. Dysbiosis of oral microbiota predicted poorer oral health, potentially contributing to chronic inflammation of the oral cavity [38]. Inflammatory substances, such as IL-6 and IL-1β, also increased in the serum of PD patients [39]. Persistent inflammation and immune response could disrupt the integrity of the blood-brain barrier, allowing bacteria to reach the brain more easily and influencing nervous system function.

Furthermore, based on predicted gene function, we demonstrated the most prominent metabolic pathway was arginine and proline metabolism at the L3-level KEGG pathways. Arginine metabolic pathways can generate nitric oxide (NO), glutamate, creatine, and many other substances [40,41]. In addition, animal studies have suggested that NO appears to participate in the modulation of panic-like behavior, as demonstrated in animal studies [42,43] and in research on patients with PD who exhibit higher serum NO levels [44,45]. Therefore, we speculated that oral microbiota may influence PD via producing NO through metabolic pathways.

Just as oral microbiota composition influences PD, the opposite also occurs. Heightened emotions and stress could activate the HPA axis, causing cortisol secretion [46,47]. Elevated oral cortisol then leads to alterations in oral microbial composition and metabolism. As an example, Fusobacteria and Leptotrichia activity improves with increasing cortisol concentrations [48]. Additionally, high cortisol concentrations promoted the growth of periodontitis-related microbiota [49] and were noted in patients with PD [50]. Together, these results suggest that PD activates the HPA axis to alter oral microbial composition. Further research should perform in-depth examinations on the mechanisms involved in oral microbiota and PD interactions.

## Limitations

This study had several limitations. First, we used a cross-sectional research design with low causal effectiveness. Second, our sample size was small and not strongly representative. Finally, we did not fully reduce confounding effects from other variables (e.g., dietary habits and regional factors). In the future, inclusion and exclusion criteria with greater completeness and detail will be necessary to address this issue.

## Conclusion

For the first time, we showed that oral microbes differed between patients with PD and healthy subjects. The effect of oral microbiota on PD appears to occur through metabolic and inflammatory pathways. The relationship is reciprocal, with PD activating the HPA axis to influence oral microbial composition and metabolism. We recommend larger prospective cohort studies to investigate the interaction between oral microbiota and PD in the future.

## Supplementary Material

Supplemental Material

## Research highlights

- Oral microbiota differed between healthy controls and patients with panic disorder.
- Oral microbiota may influence panic disorder via inflammatory and metabolic pathways.
- Oral microbiota and panic disorder may exhibit a reciprocal interaction effect.
