# Supplementary material for: Alterations of oral microbiota in patients with panic disorder
Source: Bioengineered. 2021 Oct 26;12(1):9103–12. doi: 10.1080/21655979.2021.1994738 (PMC8806997; doi:10.1080/21655979.2021.1994738)
Supplement: Supplemental Material [file KBIE_A_1994738_SM3886.docx]

**supplementary materials**

**supplementary tables**

Supplementary table S1— Core microbiota OTUs

| **OTU ID** | **Taxonomy** | **name** |
| --- | --- | --- |
| denovo26 | genus | Actinomyces |
| denovo6 | genus | Fusobacterium |
| denovo16 | genus | Gemella |
| denovo5 | genus | Granulicatella |
| denovo3 | genus | Haemophilus |
| denovo21 | genus | Lachnoanaerobaculum |
| denovo11 | genus | Lautropia |
| denovo371 | genus | Leptotrichia |
| denovo10 | genus | Neisseria |
| denovo4 | genus | Oribacterium |
| denovo17 | genus | Porphyromonas |
| denovo8 | genus | Rothia |
| denovo7 | genus | Saccharibacteria_genera_incertae_sedis |
| denovo1 | genus | Streptococcus |
| denovo18 | genus | Veillonella |

OTU, operational taxonomic unit

Supplementary table S2—Relative abundance between PD patients and healthy controls

| **Taxon name** | **MAD(PD-HC)** | ***p*-values** | **FDR** |
| --- | --- | --- | --- |
| **Phylum** |  |  |  |
| Bacteroidetes | 5.79E-02 | 0.0006 | 0.0080 |
| Candidatus Saccharibacteria | 1.31E-02 | 0.0002 | 0.0043 |
| Chloroflexi | 9.38E-05 | 0.0191 | 0.0871 |
| Deferribacteres | 3.79E-04 | 0.0045 | 0.0313 |
| Fusobacteria | -2.85E-02 | 0.0205 | 0.0893 |
| Spirochaetes | 1.33E-04 | 0.0154 | 0.0755 |
| SR1 | 1.83E-03 | 0.0010 | 0.0111 |
| Synergistetes | 2.50E-03 | 0.0006 | 0.0080 |
| Verrucomicrobia | 1.01E-02 | 0.0001 | 0.0033 |
| **Class** |  |  |  |
| Alphaproteobacteria | 1.35E-04 | 0.0030 | 0.0245 |
| Anaerolineae | 9.16E-05 | 0.0274 | 0.1149 |
| Bacteroidia | 5.32E-02 | 0.0009 | 0.0108 |
| Betaproteobacteria | -2.92E-02 | 0.0448 | 0.1570 |
| Deferribacteres | 3.79E-04 | 0.0045 | 0.0313 |
| Deltaproteobacteria | 4.43E-04 | 0.0073 | 0.0459 |
| Epsilonproteobacteria | -7.78E-03 | 0.0479 | 0.1633 |
| Erysipelotrichia | 5.56E-04 | 0.0165 | 0.0776 |
| Fusobacteriia | -2.85E-02 | 0.0205 | 0.0893 |
| Negativicutes | 4.07E-02 | 0.0000 | 0.0024 |
| Spirochaetia | 1.33E-04 | 0.0154 | 0.0755 |
| Synergistia | 2.50E-03 | 0.0006 | 0.0080 |
| Verrucomicrobiae | 1.01E-02 | 0.0001 | 0.0033 |
| **Order** |  |  |  |
| Actinomycetales | -7.40E-02 | 0.0454 | 0.1570 |
| Anaerolineales | 9.16E-05 | 0.0274 | 0.1149 |
| Bacteroidales | 5.32E-02 | 0.0009 | 0.0108 |
| Bifidobacteriales | 8.50E-04 | 0.0052 | 0.0347 |
| Campylobacterales | -7.78E-03 | 0.0479 | 0.1633 |
| Cardiobacteriales | 1.44E-03 | 0.0004 | 0.0071 |
| Coriobacteriales | 2.50E-03 | 0.0027 | 0.0233 |
| Deferribacterales | 3.79E-04 | 0.0045 | 0.0313 |
| Desulfobacterales | 2.00E-04 | 0.0378 | 0.1423 |
| Desulfovibrionales | 1.61E-04 | 0.0085 | 0.0510 |
| Enterobacteriales | -3.14E-03 | 0.0037 | 0.0284 |
| Erysipelotrichales | 5.56E-04 | 0.0165 | 0.0776 |
| Fusobacteriales | -2.85E-02 | 0.0205 | 0.0893 |
| Pseudomonadales | -6.12E-04 | 0.0147 | 0.0755 |
| Rhodobacterales | -1.31E-05 | 0.0451 | 0.1570 |
| Rhodospirillales | 9.82E-06 | 0.0392 | 0.1455 |
| Selenomonadales | 4.07E-02 | 0.0000 | 0.0024 |
| Sphingomonadales | 4.50E-05 | 0.0023 | 0.0214 |
| Spirochaetales | 1.33E-04 | 0.0154 | 0.0755 |
| Synergistales | 2.50E-03 | 0.0006 | 0.0080 |
| Verrucomicrobiales | 1.01E-02 | 0.0001 | 0.0033 |
| **Family** |  |  |  |
| Acidaminococcaceae | 1.52E-04 | 0.0003 | 0.0071 |
| Anaerolineaceae | 9.16E-05 | 0.0274 | 0.1149 |
| Bacteroidaceae | 1.31E-03 | 0.0001 | 0.0033 |
| Bacteroidales_incertae_sedis | 3.15E-04 | 0.0308 | 0.1215 |
| Bifidobacteriaceae | 8.50E-04 | 0.0052 | 0.0347 |
| Campylobacteraceae | -7.75E-03 | 0.0442 | 0.1570 |
| Cardiobacteriaceae | 1.44E-03 | 0.0004 | 0.0071 |
| Clostridiales_Incertae Sedis XI | 6.47E-04 | 0.0380 | 0.1423 |
| Clostridiales_Incertae Sedis XIII | 1.21E-03 | 0.0073 | 0.0459 |
| Comamonadaceae | 2.18E-03 | 0.0194 | 0.0877 |
| Coriobacteriaceae | 2.50E-03 | 0.0027 | 0.0233 |
| Corynebacteriaceae | 7.91E-03 | 0.0001 | 0.0033 |
| Deferribacteraceae | 3.79E-04 | 0.0045 | 0.0313 |
| Desulfobulbaceae | 2.00E-04 | 0.0378 | 0.1423 |
| Desulfovibrionaceae | 2.16E-04 | 0.0090 | 0.0533 |
| Enterobacteriaceae | -3.14E-03 | 0.0037 | 0.0284 |
| Erysipelotrichaceae | 5.56E-04 | 0.0165 | 0.0776 |
| Lactobacillaceae | 2.43E-03 | 0.0013 | 0.0134 |
| Micrococcaceae | -8.49E-02 | 0.0292 | 0.1200 |
| Moraxellaceae | 1.44E-04 | 0.0122 | 0.0671 |
| Peptococcaceae 1 | 1.38E-04 | 0.0493 | 0.1656 |
| Porphyromonadaceae | 2.18E-02 | 0.0064 | 0.0412 |
| Prevotellaceae | 2.77E-02 | 0.0007 | 0.0086 |
| Rhodobacteraceae | -1.31E-05 | 0.0451 | 0.1570 |
| Rikenellaceae | 1.61E-03 | 0.0000 | 0.0023 |
| Ruminococcaceae | 2.91E-03 | 0.0001 | 0.0033 |
| Sphingomonadaceae | 4.98E-05 | 0.0113 | 0.0627 |
| Spirochaetaceae | 1.38E-04 | 0.0154 | 0.0755 |
| Sutterellaceae | 3.95E-04 | 0.0038 | 0.0287 |
| Synergistaceae | 2.50E-03 | 0.0006 | 0.0080 |
| Veillonellaceae | 4.05E-02 | 0.0000 | 0.0024 |
| Verrucomicrobiaceae | 1.01E-02 | 0.0001 | 0.0033 |
| **Genus** |  |  |  |
| Acetatifactor | 1.05E-04 | 0.0059 | 0.0383 |
| Acinetobacter | 1.29E-04 | 0.0111 | 0.0627 |
| Aggregatibacter | 1.14E-04 | 0.0049 | 0.0337 |
| Akkermansia | 1.01E-02 | 0.0001 | 0.0033 |
| Alistipes | 1.61E-03 | 0.0000 | 0.0023 |
| Alloprevotella | 7.90E-04 | 0.0009 | 0.0105 |
| Anaeroglobus | 3.27E-05 | 0.0196 | 0.0878 |
| Anaerovorax | 1.11E-03 | 0.0152 | 0.0755 |
| Atopobium | 1.01E-03 | 0.0446 | 0.1570 |
| Bacteroides | 1.31E-03 | 0.0001 | 0.0033 |
| Barnesiella | 8.96E-04 | 0.0005 | 0.0079 |
| Bifidobacterium | 4.80E-04 | 0.0023 | 0.0214 |
| Blautia | 2.37E-05 | 0.0109 | 0.0627 |
| Campylobacter | -7.74E-03 | 0.0442 | 0.1570 |
| Capnocytophaga | 5.01E-03 | 0.0042 | 0.0313 |
| Cardiobacterium | 1.44E-03 | 0.0004 | 0.0071 |
| Clostridium IV | 1.68E-04 | 0.0018 | 0.0178 |
| Clostridium XlVa | 1.33E-04 | 0.0075 | 0.0463 |
| Coprococcus | 1.50E-05 | 0.0080 | 0.0486 |
| Corynebacterium | 7.91E-03 | 0.0001 | 0.0033 |
| Desulfobulbus | 2.00E-04 | 0.0378 | 0.1423 |
| Dialister | 1.94E-04 | 0.0001 | 0.0033 |
| Escherichia/Shigella | -2.63E-03 | 0.0011 | 0.0114 |
| Faecalibacterium | 3.33E-04 | 0.0033 | 0.0265 |
| Flavonifractor | 5.70E-05 | 0.0175 | 0.0817 |
| Fretibacterium | 2.49E-03 | 0.0007 | 0.0083 |
| Gemmiger | 7.18E-05 | 0.0225 | 0.0969 |
| Lachnospiracea_incertae_sedis | 3.79E-04 | 0.0000 | 0.0026 |
| Lactobacillus | 2.44E-03 | 0.0013 | 0.0134 |
| Megamonas | 6.36E-04 | 0.0132 | 0.0716 |
| Meganema | 4.81E-06 | 0.0305 | 0.1215 |
| Megasphaera | 6.78E-04 | 0.0027 | 0.0233 |
| Mucispirillum | 3.79E-04 | 0.0045 | 0.0313 |
| Odoribacter | 2.25E-04 | 0.0138 | 0.0743 |
| Oligella | -8.96E-05 | 0.0414 | 0.1526 |
| Olsenella | 2.79E-04 | 0.0029 | 0.0239 |
| Oscillibacter | 1.83E-04 | 0.0025 | 0.0230 |
| Parabacteroides | 2.90E-04 | 0.0022 | 0.0214 |
| Paracoccus | -1.92E-05 | 0.0443 | 0.1570 |
| Parasutterella | 3.91E-04 | 0.0028 | 0.0239 |
| Parvimonas | 3.38E-04 | 0.0344 | 0.1334 |
| Peptococcus | 1.38E-04 | 0.0493 | 0.1656 |
| Phascolarctobacterium | 1.52E-04 | 0.0004 | 0.0071 |
| Phocaeicola | 3.15E-04 | 0.0308 | 0.1215 |
| Prevotella | 2.65E-02 | 0.0005 | 0.0079 |
| Psychrobacter | 1.90E-05 | 0.0306 | 0.1215 |
| Romboutsia | 3.76E-05 | 0.0337 | 0.1317 |
| Roseburia | 3.29E-04 | 0.0005 | 0.0079 |
| Rothia | -8.49E-02 | 0.0292 | 0.1200 |
| Ruminococcus | 9.75E-05 | 0.0004 | 0.0071 |
| Saccharibacteria_genera_incertae_sedis | 1.31E-02 | 0.0002 | 0.0043 |
| Schwartzia | 2.17E-03 | 0.0096 | 0.0561 |
| Selenomonas | 1.20E-02 | 0.0006 | 0.0080 |
| Shuttleworthia | 1.64E-04 | 0.0160 | 0.0776 |
| Sphingomonas | 3.80E-05 | 0.0113 | 0.0627 |
| SR1_genera_incertae_sedis | 1.83E-03 | 0.0010 | 0.0111 |
| Tannerella | 3.77E-04 | 0.0182 | 0.0842 |
| Treponema | 1.41E-04 | 0.0154 | 0.0755 |
| Turicibacter | 2.98E-04 | 0.0142 | 0.0753 |
| Veillonella | 2.48E-02 | 0.0001 | 0.0035 |
| Weeksella | -2.41E-04 | 0.0306 | 0.1215 |

PD, panic disorder; HC, healthy controls;

MAD, mean abundance difference;

FDR, false discovery rate

Supplementary table S3 —KOs with differences between PD patients and healthy controls

| **KO identifier** | **Enrich group** | **LDA score** | ***p*-value** |
| --- | --- | --- | --- |
| K14645 | HC | 2.027502 | 0.012653 |
| K00655 | HC | 2.084626 | 0.005401 |
| K07448 | HC | 2.019377 | 0.013128 |
| K07118 | HC | 2.059526 | 0.00478 |
| K02028 | HC | 2.201546 | 0.023997 |
| K00014 | HC | 2.010913 | 0.016327 |
| K07270 | HC | 2.016048 | 0.013128 |
| K02484 | HC | 2.032966 | 0.001788 |
| K02302 | HC | 2.245871 | 0.005401 |
| K14260 | HC | 2.088587 | 0.002231 |
| K11737 | HC | 2.005002 | 0.01575 |
| K02013 | HC | 2.125816 | 0.010497 |
| K02483 | HC | 2.028358 | 5.93E-05 |
| K02030 | HC | 2.202001 | 0.017536 |
| K02035 | HC | 2.429628 | 0.006093 |
| K03303 | HC | 2.268631 | 0.018822 |
| K00004 | HC | 2.074941 | 0.005623 |
| K09940 | HC | 2.110196 | 0.034616 |
| K00974 | HC | 2.200846 | 0.000141 |
| K05366 | PD | 2.051395 | 0.001084 |
| K07114 | PD | 2.021218 | 0.000166 |
| K03561 | PD | 2.100862 | 0.00071 |
| K03296 | PD | 2.021998 | 0.00071 |
| K03088 | PD | 2.581641 | 7.40E-05 |
| K06142 | PD | 2.068742 | 0.00034 |
| K03924 | PD | 2.074541 | 7.81E-05 |
| K03797 | PD | 2.014105 | 0.001365 |
| K00266 | PD | 2.122342 | 0.000531 |
| K13789 | PD | 2.080604 | 0.004054 |

KO, KEGG orthology; KEGG, Kyoto Encyclopedia of Genes and Genomes;

PD, panic disorder; HC, healthy controls; LDA, Linear discriminant analysis
